# Variability of the retrotympanum and its association with mastoid pneumatization in cholesteatoma patients

**DOI:** 10.1007/s00405-022-07465-w

**Published:** 2022-06-13

**Authors:** Sara-Lynn Hool, Sven Beckmann, Arsany Hakim, Abraam Yacoub, Marco Caversaccio, Franca Wagner, Lukas Anschuetz

**Affiliations:** 1 Department of Otorhinolaryngology, Head and Neck Surgery, Inselspital, University Hospital and University of Bern, Freiburgstrasse 15, 3010 Bern, Switzerland; 2 Department of Diagnostic and Interventional Neuroradiology, Inselspital, University Hospital and University of Bern, Bern, Switzerland; 3 Department of Otolaryngology Head and Neck Surgery, Faculty of Medicine, Ain Shams University, Cairo, Egypt

**Keywords:** Retrotympanum, Cholesteatoma, Temporal bone anatomy, Pneumatization, Endoscopic ear surgery

## Abstract

**Purpose:**

This study aimed to investigate the variability of the retrotympanum in patients undergoing surgical treatment for cholesteatoma.

**Methods:**

We included 59 ears of patients undergoing middle ear surgery for cholesteatoma who had preoperative computed tomography scans. A retrospective analysis of the medical records was conducted. The sinus tympani (ST), subtympanic sinus (STS) and facial recess (FR) were classified into types A–C based on the relationship of their extension to the facial nerve. The mastoid and petrous apex were assessed and categorized as normal pneumatized or sclerotic.

**Results:**

Type A extension was the most frequently found in all sinuses (ST 64%, FR 77%, STS 69%), Type B extension was found more often in ST (34%) and STS (24%) than in FR (15%). A very deep extension was found only rarely (ST 2%, FR 8%, STS 7%). A sclerotic mastoid was found in 67% of cases. Those cases showed a statistically significant difference regarding retrotympanum pneumatization when compared with normal mastoid.

**Conclusion:**

The most frequent variant of retrotympanic pneumatization in relation to the facial nerve was type A in all subsites in cholesteatoma patients. The variability among patients with cholesteatoma is different to previously published results in healthy subjects. Moreover, the pneumatization of the retrotympanum is associated with mastoid pneumatization.

## Introduction

**Fig. 1:**
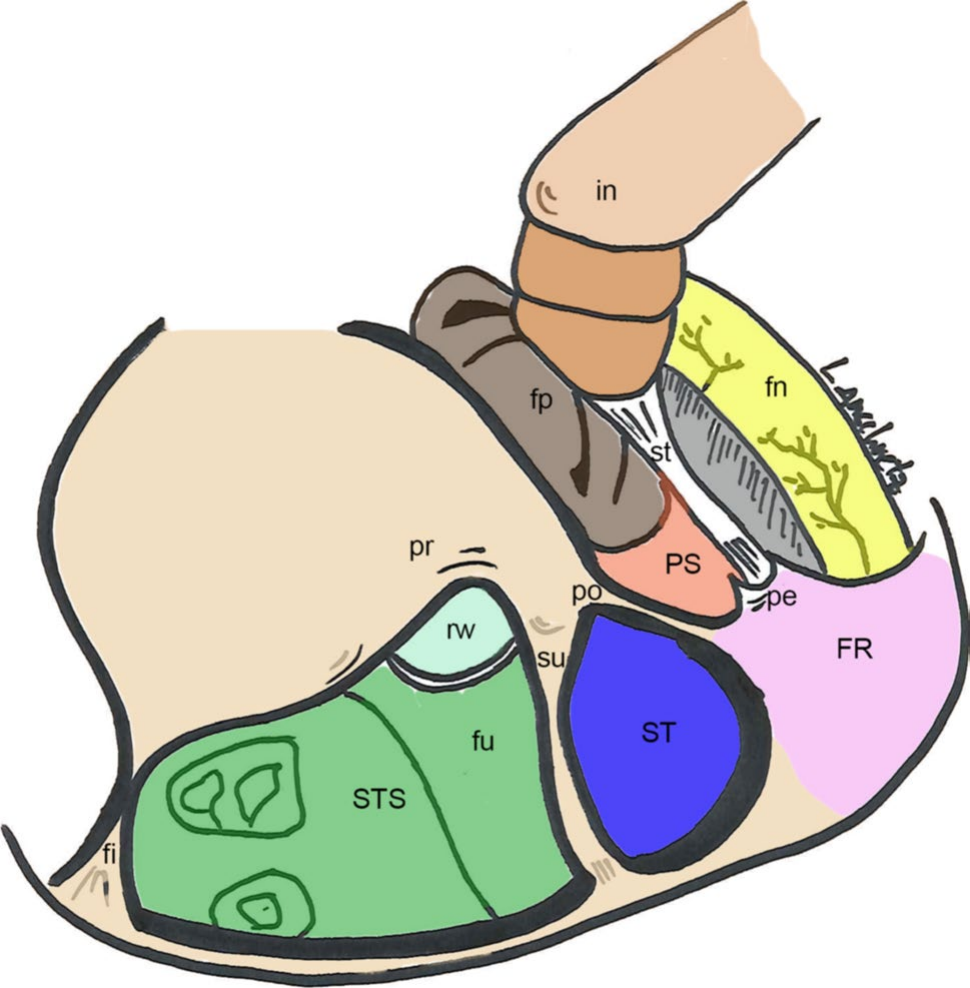
Left ear: Schematic illustration of the anatomy of the retrotympanum. *In* incus, *fp* footplate, *fn* facial nerve, *pr* promontory, *PS* posterior sinus, *po* ponticulus, *su* subiculum, *pe* pyramidal eminence, *rw* round window, *FR* facial recess, *ST* sinus tympani, *fu* fustis, *STS* subtympanic sinus, *fi* finiculus

The retrotympanum represents the posterior wall of the tympanic cavity and hosts important anatomical structures, such as the facial nerve and the round window niche [1]. It is characterized by multiple bony bridges and sinuses, which show marked complexity and variability, as illustrated in Fig. 1. Moreover, middle ear diseases—especially cholesteatoma—frequently involve the retrotympanum [2]. Therefore, this hidden region of the middle ear plays an important role when performing surgical treatment of middle ear cholesteatoma. Owing to its localization and posterior extension, cholesteatoma removal is often surgically challenging. Indeed, several authors have shown that persistence of cholesteatoma is frequent in this region [3–5]. Accordingly, knowledge of the anatomy, its variability and the identification of difficult variants on preoperative computed tomography (CT) scans are important for planning and performing surgery.

The introduction of the endoscope for middle ear surgery allowed a greater insight into this hidden region, and endoscopic and radiologic classifications of subsites have recently been published. The current groupings classify the pneumatization of the sinus tympani (ST), sinus subtympanicus (STS) and facial recess (FR) in relation to the facial nerve as follows [6–9]:

- *Type A* no extension of the sinus in relation to the level of the facial nerve.
- *Type B* extension of the sinus posterior to the level of the facial nerve.
- *Type C* extension medial and posterior to the level of the facial nerve.

**Fig. 2:**
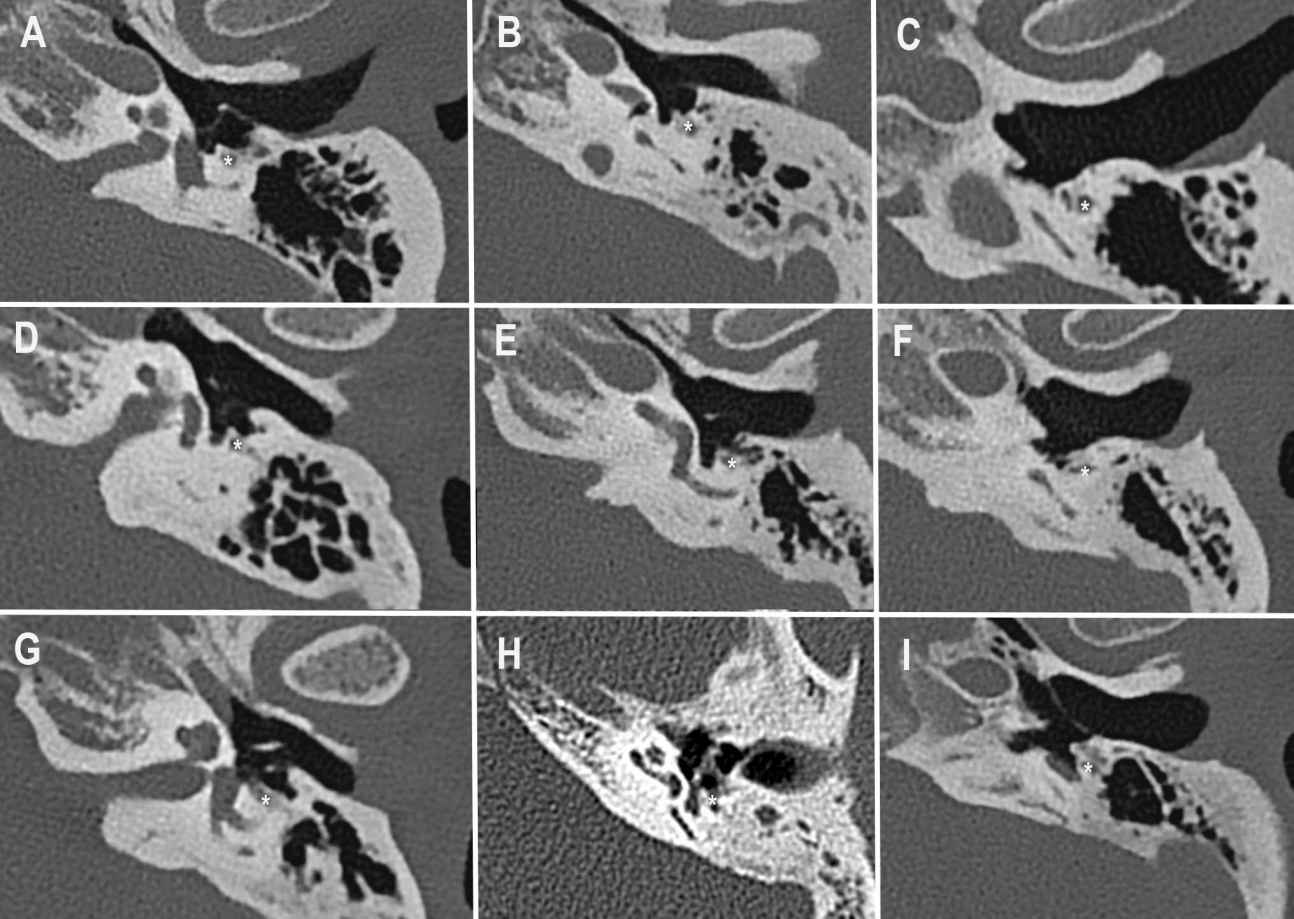
Radiologic classification of retrotympanum in Type A/B/C: Facial recess (**A**/**D**/**G**), sinus tympani (**B**/**E**/**H**) and subtympanic sinus (**C**/**F**/**I**). *Facial nerve

The radiological presentation of these different types is illustrated in Fig. 2.

However, a recent comprehensive comparison between endoscopic visibility and radiologic extension revealed the limitations of endoscopic exploration of extensively pneumatized retrotympanic subsites [8, 9]. Even the use of 70° angled lenses did not permit the complete exploration of the sinus in cases of extensive pneumatization [9].

Furthermore, most classifications rely on data collected in healthy specimens of temporal bone. In contrast, most ears affected by cholesteatoma show some degree of reduced pneumatization [10]. We therefore hypothesized a difference in the anatomical variability of diseased ears as compared to the healthy specimens as reported in the literature.

## Methods

### Ethical issues

The local ethics committee reviewed and approved the present study (Kantonale Ethikkommission Bern 2019-00555) which was performed according to the Declaration of Helsinki [11].

### Patients

Medical records from our tertiary referral center were retrospectively analyzed and details of patients undergoing endoscopic surgery of the middle ear for cholesteatoma were extracted (*n* = 84). Endoscopic cholesteatoma infiltration was based on reviewed surgical reports and videos. In cases of extension of the cholesteatoma to the mastoid, an additional retroauricular microscopic approach—canal wall up (CWU) was performed. In extensive cases with infiltration of the external auditory canal, lateral semicircular canal fistula or infiltration of the internal auditory canal, a canal wall down (CWD) tympanomastoidectomy was performed to completely eradicate the disease. For inclusion in the present study, patients had to have histological confirmation of cholesteatoma and a preoperative high-resolution temporal bone CT scan. Patients undergoing revision surgery for cholesteatoma were excluded (*n* = 16) as were patients without suitable preoperative imaging (*n* = 9).

### Image acquisition

All in-house CT scans were acquired using our 128-slice CT scanner (SOMATOM1 Definition Edge; Siemens Healthcare, Erlangen, Germany). A certified reporting workstation (Sectra IDS7, Linköping, Sweden) was used for evaluation by the 2 neuroradiologists, who were blinded to the clinical data. All the images collected were of sufficiently good quality to allow an accurate assessment of the middle ear. The standard in-house temporal bone CT scan parameters (Somatom Edge, Siemens, Erlangen, Germany) are as follows: slice thickness (SL) 0.6.mm, field of view (FoV) 160 mm, total acquisition time of 1 s by tube current–time product of 230 mA, and tube voltage 120 kV. This resulted on average in a computed tomography dose index (CTDI) of 35 mGy and a dose–length product (DLP) of 330mGycm.

Image reconstruction according to our standard in-house temporal bone protocol included a soft tissue window (kernel J45s) and a bone window (kernel J70h) of the acquired CT scans, each in the axial, coronal, and an oblique (Stenvers) plane. For our retrospective data analysis, both neuroradiologists additionally reconstructed the CT scans in the multiplanar reconstruction mode (MPR-mode) to provide a different view of the ossicular chain, the retrotympanum and middle ear cavity. Both 2D and MPR3 were used for evaluation.

The external CTs of the temporal bone were scanned with a maximum of a SL 1 mm and a minimum of a SL 0.4 mm, the FoV differed between 150 and 250 mm with resulting range of exposure parameters.

### Image analysis

Two board-certified neuroradiologists with more than 10 years of experience separately performed blinded imaging analysis. According to the classification described above, the tympanic sinus, subtympanic sinus and facial recess were classified as type A, B or C. After a first independent rating, a consensus meeting was held to review all cases subject to disagreement after which consensus was reached on all cases, resulting in a single rating per subsite. In addition, the mastoid part of the temporal bone was examined and classified: “normal pneumatization” for good pneumatization or hyperpneumatization and “sclerotic” for moderate pneumatization or hypopneumatization according to Dexian Tan et al. [12]. The petrous apex was examined and classified: “pneumatized” for pneumatized air cells medial to the labyrinth or “not pneumatized” for no air cells in the proximity of the inner ear as described by Dexian Tan et al. (2018). The data were analyzed using GraphPad Prism 9.2 (La Jolla, USA). Comparative statistics were performed with a two-tailed *α* set to < 0.05.

## Results

Fifty-four patients were enrolled in the present study with a total of 59 affected ears. Of these, 56% were left ears. All patients underwent preoperative high-resolution CT scanning of the temporal bone, with a mean slice thickness of 0.63 mm (range 0.3–1 mm). The chosen surgical approach was exclusively endoscopic in 36 patients (61%), combined (endoscopic for middle ear and microscopic for mastoid surgery) CWU in 17 (29%) and combined CWD in 6 patients (10%). An endoscopic infiltration of the retrotympanum was observed in 30 ears (51%), requiring endoscopic cholesteatoma removal.

**Fig. 3:**
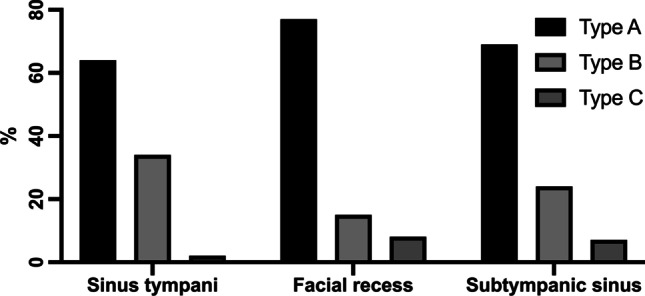
Radiologic distribution of retrotympanic sinus configuration in cholesteatoma cases

After the first round of image analysis and classification of the retrotympanic sinus into Type A, B or C by the experienced neuroradiologists as described above, there was disagreement of 49% for sinus tympani, 47% for facial recess and 24% for subtympanic space. A consensus meeting was held during which the retrotympanic spaces were re-classified. Finally, a consensus was achieved in all cases. Type A extension was most often observed in all sinuses. Type B extension was seen most often in the sinus tympani (34%). A very deep extension medial and posterior to the facial nerve was only rarely observed in any of the subsites, as summarized in Table 1 and illustrated in Fig. 3. Poor mastoid pneumatization was observed in 67% of the cases and the petrous apex was pneumatized in only 1 case.

**Table 1:** Radiologic classification of the retrotympanum in cholesteatoma cases

	Type A	Type B	Type C
Sinus tympani	38 (64%)	20 (34%)	1 (2%)
Facial recess	45 (77%)	9 (15%)	5 (8%)
Subtympanic sinus	41 (69%)	14 (24%)	4 (7%)

**Fig. 4:**
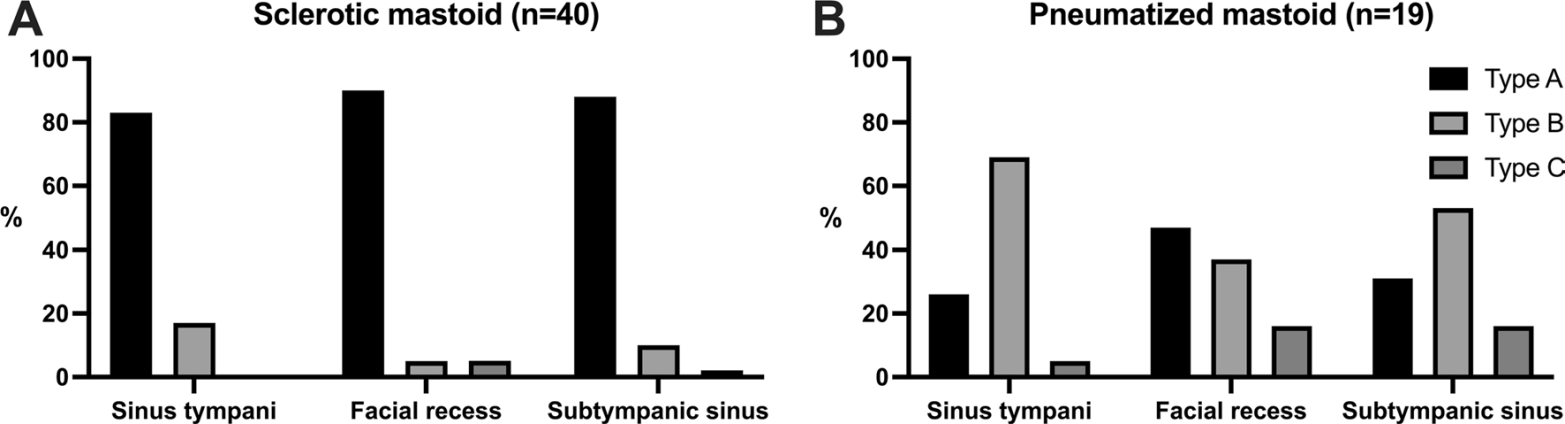
**A** Association of sclerotic mastoid and retrotympanum pneumatization. Type A configuration of all sinuses was found statistically significantly more often than in normal pneumatized mastoid. **B** Distribution of retrotympanum pneumatization in normal mastoid

Comparison between cases with a sclerotic mastoid and those with a pneumatized mastoid revealed a statistically significant difference in pneumatization of the retrotympanum (*p* < 0.001). This association is illustrated in Fig. 4. No association between presence of disease in the retrotympanum and its anatomical variability was observed.

## Discussion

This study investigated the variability of the retrotympanum in patients undergoing surgical treatment for cholesteatoma. The preoperative radiological analysis revealed the highest anatomical variability regarding extension of the retrotympanum in relation to the facial nerve in the sinus tympani. A type A extension was most common at all subsites. Patients with a sclerotic mastoid, indicating long-standing impairment of ventilation of the mastoid and the middle ear showed a significant difference in pneumatization as compared to patients with normal mastoid pneumatization. Therefore, an association between mastoid and retrotympanum pneumatization may be hypothesized on the basis of our results.

The retrotympanum is of particular interest to surgeons treating patients with chronic middle ear diseases and especially those with cholesteatoma. It is well known that the retrotympanum is the most frequent site of postoperative disease persistence [9], leading to continuous expansion of the cholesteatoma and a requirement for revision surgery. Therefore, a thorough knowledge of retrotympanic anatomy, appropriate preoperative planning and complete cholesteatoma removal are of the utmost importance in ensuring the best surgical outcome. The sinus tympani has been classified as a difficult access site in the most recent classification by the European and Japanese Otological Societies [13]. The introduction of endoscopes led to considerable advances in the surgical management of the retrotympanum allowing full exploration and cholesteatoma removal in most cases, as reported by Marchioni et al. [6]. So far, this is the only report on the morphological variability of the retrotympanum in patients with chronic middle ear disease. The subsequently published results and classifications of the retrotympanum were based on studies on cadavers of healthy donors [7, 8, 14]. The currently available results on retrotympanic variability are summarized in Table 2.

**Table 2:** Results of radiological classification of the retrotympanum in the current literature

	Type A	Type B	Type C
Definition [6–9]	No extension of the sinus in relation to the level of the facial nerve	Extension of the sinus posterior or medial (for STS) to the facial nerve	Extension of the sinus posterior and medial to the facial nerve
Sinus tympani	– 47% (*n* = 14) Bonali et al. [9]	– 47% (*n* = 14) Bonali et al. [9]	– 6% (*n* = 2) Bonali et al. [9]
	– 45% (*n* = 14) Alicandri-Ciufelli et al. [7]	– 48% (*n* = 15) Alicandri-Ciufelli et al. [7]	– 7% (*n* = 2) Alicandri-Ciufelli et al. [7]
	– 66% (*n* = 82) Bonali et al. [14]	– 26% (*n* = 33) Bonali et al. [14]	– 6% (*n* = 8) Bonali et al. [14]
	– 35% (*n* = 14)^a^ Marchioni et al. [6]	– 52.5% (*n* = 21)^a^ Marchioni et al. [6]	– 12.5% (*n* = 5)^a^ Marchioni et al. [6]
Facial recess	– 60% (*n* = 18) Bonali et al. [9]	– 27% (*n* = 8) Bonali et al. [9]	– 13% (*n* = 4) Bonali et al. [9]
	– 58% (*n* = 18) Alicandri-Ciufelli et al. [7]	– 29% (*n* = 9) Alicandri-Ciufelli et al. [7]	– 13% (*n* = 4) Alicandri-Ciufelli et al. [7]
Subtympanic sinus	– 46.5% (*n* = 14) Bonali et al. [9]	– 46.5% (*n* = 14) Bonali et al. [9]	– 6% (*n* = 2) Bonali et al. [9]
	– 72% (*n* = 21) Anschuetz et al. [8]	– 21% (*n* = 6) Anschuetz et al. [8]	– 7% (*n* = 2) Anschuetz et al. [8]
Summary			
Total *n* = 304^b^	59.5% (*n* = 181)	32.5% (*n* = 99)	8% (*n* = 24)

Except for Marchioni et al. [6], all data related to healthy ears. The results of Marchioni et al. [6] were therefore excluded from the summary totals

*STS* subtympanic sinus

^a^Patients with middle ear disease

^b^Excluding Marchioni et al. [6]

When comparing these data to our results in patients undergoing surgery for cholesteatoma, we observed a higher prevalence of Type A configuration in all retrotympanic subsites as follows: sinus tympani: 64% (literature: 52.5%), facial recess: 77% (literature: 59%) and subtympanic sinus: 69% (literature: 59%). Overall, Type A configuration was observed in 70% of cholesteatoma cases as compared to 59.5% in healthy ears.

Recently, Bonali et al. [9] investigated the correlation of radiologic configuration of the retrotympanic subsites to the visibility of the medial and posterior walls during a transcanal endoscopic approach. They revealed limitations of the endoscopic approach in deeply pneumatized retrotympanic spaces in healthy body donors. Even using 70° lenses it was not possible fully visualize a type C facial recess and subtympanic sinus in as many as 50% of the investigated ears. The sinus tympani was fully explorable using 70° lenses even in type C variants. This is clearly relevant to the surgical treatment of cholesteatoma in deeply pneumatized retrotympanic spaces. The surgeon needs to identify such cases preoperatively and, where necessary, to be prepared to perform a retrofacial approach to the middle ear to remove the cholesteatoma completely. However, according to our results, these deeply pneumatized variants appear to be even less frequent than previously reported. Interestingly, impaired pneumatization of the mastoid appeared to be associated with a less pneumatized retrotympanum in our cohort, when comparing cholesteatoma patients with sclerotic mastoid spaces to those with pneumatized ones. These findings are in line with a recently published study which found the pneumatization of the mastoid of patients with chronic otitis media was associated with the depth of the sinus tympani only [15]. Our study found that this association applied to the facial recess and the subtympanic sinus as well. However, involvement of the retrotympanum by cholesteatoma was not associated with its pneumatization.

Although our findings relevant to preoperative planning based on the high-resolution CT scans are important, image analysis and classification is challenging. In our experience, this applies even to expert neuroradiologists with many years of experience and; in our study, there was disagreement in many cases, requiring resolution at a consensus meeting. However, re-examination of the CT scans finally resulted in consensus regarding all cases. This finding is exemplary of the need for ear surgeons to be familiar with this kind of anatomy not only from a surgical, but also from a radiological perspective in order to correctly assess and interpret preoperative CT findings. In our opinion, it is important for every ear surgeon to interpret the CT images independently, especially if no experienced and appropriately trained neuroradiologists are available, otherwise important findings on the retrotympanum could be simply overlooked.

However, there are certain limitations of this study. One of the limitations of our study is its retrospective character and the lack of uniform CT scans of the temporal bone during the time period of CT acquisition. In addition, we are aware that the interpretation of a temporal bone CT, with its complex anatomy and microstructures, depends on the experience of the reader. Furthermore, previous description of anatomical variants of retrotympanic subsites were conducted in healthy cadaver specimens, leading to limited comparability with patients with chronic middle ear diseases.

## Conclusion

In cholesteatoma patients, the most frequent variant of retrotympanic pneumatization in relation to the facial nerve is type A at all subsites. The variability of patients with disease is different to previously published results of studies in healthy subjects. Moreover, the pneumatization of the retrotympanum is associated with mastoid pneumatization. These findings may be especially important for preoperative planning of cholesteatoma resection in this difficult to access area of the middle ear.

## References

[1] Nogueira JF, Mattioli F, Presutti L, Marchioni D (2013). Endoscopic anatomy of the retrotympanum. Otolaryngol Clin N Am.

[2] Palva T (1990). The pathogenesis and treatment of cholesteatoma. Acta Otolaryngol.

[3] James AL, Cushing S, Papsin BC (2016). Residual cholesteatoma after endoscope-guided surgery in children. Otol Neurotol.

[4] Thomassin JM, Korchia D, Doris JM (1993). Endoscopic-guided otosurgery in the prevention of residual cholesteatomas. Laryngoscope.

[5] Weiss MH, Parisier SC, Han JC, Edelstein DR (1992). Surgery for recurrent and residual cholesteatoma. Laryngoscope.

[6] Marchioni D, Mattioli F, Alicandri-Ciufelli M, Presutti L (2009). Transcanal endoscopic approach to the sinus tympani: a clinical report. Otol Neurotol.

[7] Alicandri-Ciufelli M, Fermi M, Bonali M (2018). Facial sinus endoscopic evaluation, radiologic assessment, and classification. Laryngoscope.

[8] Anschuetz L, Alicandri-Ciufelli M, Bonali M (2018). Novel Surgical and radiologic classification of the subtympanic sinus: implications for endoscopic ear surgery. Otolaryngol Head Neck Surg.

[9] Bonali M, Fermi M, Alicandri-Ciufelli M (2020). Correlation of radiologic versus endoscopic visualization of the middle ear: implications for endoscopic ear surgery. Otol Neurotol.

[10] Sato Y, Nakano Y, Takahashi S, Ikarashi H (1990). Suppressed mastoid pneumatization in cholesteatoma. Acta Otolaryngol Suppl.

[11] World Medical A (2013). World Medical Association Declaration of Helsinki: ethical principles for medical research involving human subjects. JAMA.

[12] Dexian Tan A, Ng JH, Lim SA, Low DYM, Yuen HW (2018). Classification of temporal bone pneumatization on high-resolution computed tomography: prevalence patterns and implications. Otolaryngol Head Neck Surg.

[13] Yung M, Tono T, Olszewska E (2017). EAONO/JOS Joint Consensus Statements on the definitions, classification and staging of middle ear cholesteatoma. J Int Adv Otol.

[14] Bonali M, Anschuetz L, Fermi M (2017). The variants of the retro- and hypotympanum: an endoscopic anatomical study. Eur Arch Otorhinolaryngol.

[15] Baklaci D, Kuzucu I, Guler I (2019). Effect of mastoid bone pneumatization on the conformation and depth of the sinus tympani, a high-resolution computed tomography study. Surg Radiol Anat.

